# Acute fatty liver of pregnancy simulating liver tumor

**DOI:** 10.1002/ccr3.1293

**Published:** 2017-11-28

**Authors:** Benjamin Anon, Béatrice Scotto, Yannick Bacq

**Affiliations:** ^1^ Department of Hepatology and Gastroenterology University Hospital of Tours Tours France; ^2^ Department of Radiology University Hospital of Tours Tours France

**Keywords:** Computed tomography, nodule, ultrasonography

## Abstract

Acute fatty liver of pregnancy (AFLP) is a rare liver disease unique to pregnancy that can lead to acute liver failure. Clinicians must have a high index of suspicion for AFLP because only early diagnosis and prompt delivery improve maternal and fetal prognosis.

A 26‐year‐old primiparous patient was admitted at 37‐weeks’ gestation for abdominal pain, nausea, and vomiting. Clinical examination was normal, and there was no hypertension or proteinuria. Body mass index prior to pregnancy was normal. Laboratory tests revealed increased serum aminotransferase (AST 206 IU/L, ALT 152 IU/L), hypoglycemia (2.20 mmol/L), hyperuricemia (445 *μ*mol/L), thrombocytopenia (146 G/L), and leukocytosis (19.8 G/L). LDH was normal (193 UI/L). Ultrasonography (US) of the liver showed one hyperechogenic nodule suggesting a tumor. The patient gave birth spontaneously to a healthy girl 1 day after admission. Computed tomography (CT) after delivery showed a well‐limited zone corresponding to the US hepatic nodule (Fig. 1). The values of liver density were lower than the spleen suggesting fatty infiltration (Fig. 2). Symptoms and biological abnormalities improved spontaneously, and CT performed 7 days after delivery showed the improvement of lesions (Fig. 3). No other cause of liver disease was found, and a diagnosis of acute fatty liver of pregnancy (AFLP) was made based on clinical, biological, and radiological findings 1.

**Figure 1:**
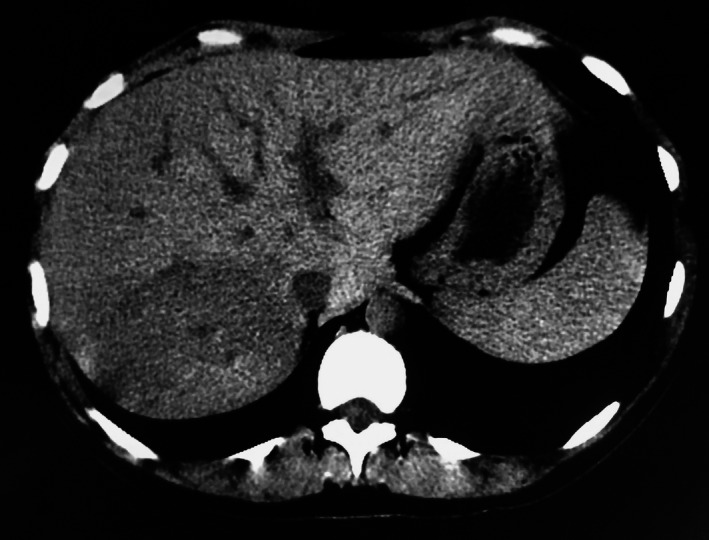
CT after delivery : showed a well‐limited zone corresponding to the US hepatic nodule.

**Figure 2:**
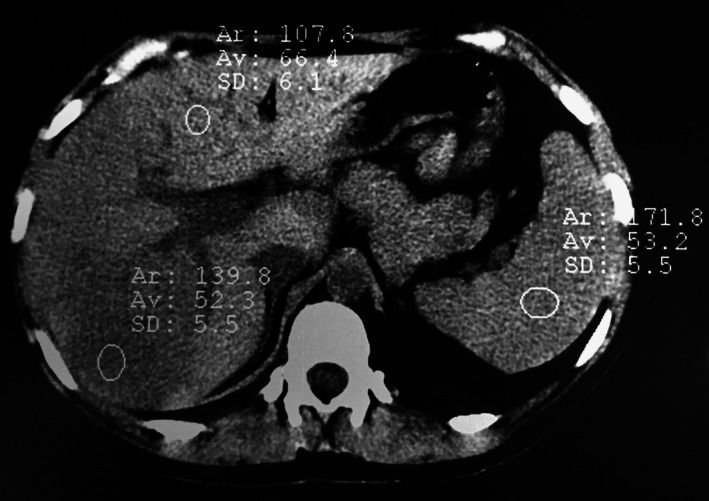
CT after delivery : the values of liver density were lower than the spleen suggesting fatty infiltration..

**Figure 3:**
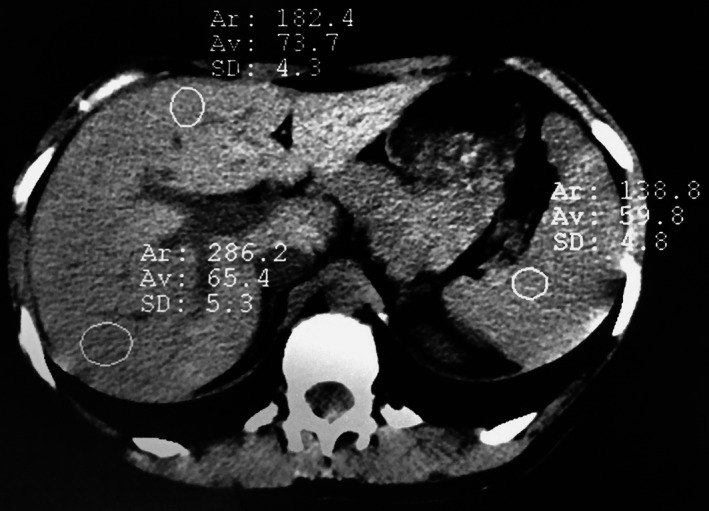
CT 7 days after delivery : showed improvement of lesions.

Clinicians must have a high index of suspicion for AFLP. Indeed, early diagnosis and prompt delivery have improved maternal and fetal prognosis of this rare liver disease unique to pregnancy 2. Moreover, AFLP may be associated with a genetic deficiency of mitochondrial beta‐oxidation of fatty acids, and screening for such deficiency is recommended for mother and baby 2. This unusual case demonstrated that AFLP, as other causes of steatosis, may mimic liver tumor at imaging.

## Authorship

BA and YB: drafted the article. BS: participated in critical review and revision of the article, gave the final approval of the article.

## Conflict of Interest

The authors have no conflict of interest to declare.

## References

[1] Ch'ng, C. L. , M. Morgan , I. Hainsworth , and J. G. C. Kingham . 2002 Prospective study of liver dysfunction in pregnancy in Southwest Wales. Gut 51:876–880. 12427793 10.1136/gut.51.6.876 PMC1773454

[2] Ibdah, J. A. 2006 Acute fatty liver of pregnancy: an update on pathogenesis and clinical implications. World J. Gastroenterol. 12:7397–7404. 17167825 10.3748/wjg.v12.i46.7397 PMC4087582

